# Outcomes of incomplete and complete revascularization in ACS

**DOI:** 10.1186/cc12174

**Published:** 2013-03-19

**Authors:** L Sayagues, J Sieira, E Abbu Assi, J Chico, C Pena, J Gonzalez Juanatey

**Affiliations:** 1 HULA, Lugo, Spain; 2 CHUS, Santiago de Compostela, Spain; 3 CHUVI, Vigo, Spain

## Introduction

This is a prospective study in which all ACS cases attending a level 3 hospital were collected consecutively from February 2004 to 2010, and were clinically followed-up until 2012.

## Methods

Of a total 4,589 cases, only 2,515 were revascularized with ICP (1,742 complete and 907 incomplete; 160 failed). We split the cases into two main groups: STEMI and NSTEMI.

## Results

**Figure 1:**
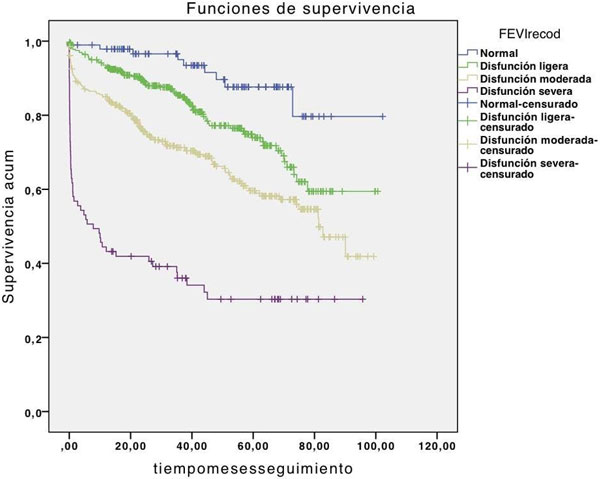
**FEVI and prognosis**.

**Figure 2:**
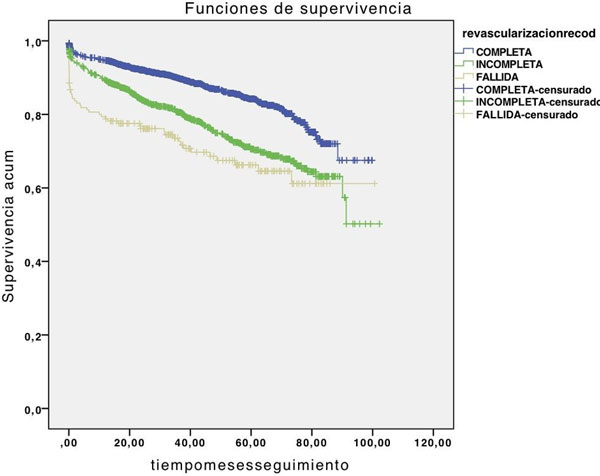
**Incomplete and complete revascularization**.

We observed a 0.6 higher accumulative survival rate in patients with complete ICP over patients who underwent surgery, incomplete ICP or mixed treatment. Those with conservative measures solely have, obviously, worst prognosis. See Figures 1 and 2.

## Conclusion

In our study patients undergoing ICP have higher survival rates in comparison with cardiac surgery except those >65 years old and diabetic groups in which cardiac surgery has higher ratios than ICP. In the rest of the groups, no matter how many coronary arteries were affected, only those with complete ICP present higher survival rates. It would be important to repeat this study in a multicentric cohort.

